# An optimized procedure for preparation of conditioned medium from Wharton’s jelly mesenchymal stromal cells isolated from umbilical cord

**DOI:** 10.3389/fmolb.2023.1273814

**Published:** 2023-10-02

**Authors:** Santina Acuto, Melania Lo Iacono, Elena Baiamonte, Rosa Lo Re, Aurelio Maggio, Vincenzo Cavalieri

**Affiliations:** ^1^ Campus of Haematology Franco e Piera Cutino, Villa Sofia-Cervello Hospital, Palermo, Italy; ^2^ Laboratory of Molecular Biology, Department of Biological, Chemical and Pharmaceutical Sciences and Technologies (STeBiCeF), University of Palermo, Palermo, Italy

**Keywords:** conditioned medium, Wharton’s jelly, mesenchymal stromal cells, umbilical cord, secreted factor profiling, human platelet lysate, chemoattractant, cytoprotective effect

## Abstract

Cell-free therapy based on conditioned medium derived from mesenchymal stromal cells (MSCs) has gained attention in the field of protective and regenerative medicine. However, the exact composition and properties of MSC-derived conditioned media can vary greatly depending on multiple parameters, which hamper standardization. In this study, we have optimized a procedure for preparation of conditioned medium starting from efficient isolation, propagation and characterization of MSCs from human umbilical cord, using a culture medium supplemented with human platelet lysate as an alternative source to fetal bovine serum. Our procedure successfully maximizes the yield of viable MSCs that maintain canonical key features. Importantly, under these conditions, the compositional profile and biological effects elicited by the conditioned medium preparations derived from these MSC populations do not depend on donor individuality. Moreover, approximately 120 L of conditioned medium could be obtained from a single umbilical cord, which provides a suitable framework to produce industrial amounts of toxic-free conditioned medium with predictable composition.

## 1 Introduction

Over the past three decades, MSCs have become the most frequently used stem cell population in the field of cellular therapy and tissue engineering (Murphy et al., 2013; Han et al., 2019; Levy et al., 2020). MSCs are multipotent stem cells that can be isolated from different sources, including bone marrow, adipose tissue, dental pulp, umbilical cord, placenta, and amnion. Thanks to their low immunogenic properties, self-renewal, and multiple differentiation abilities, MSCs emerged as a pivotal therapeutic tool in cell therapy of degenerative, immune system, gastrointestinal, musculoskeletal, and vascular diseases, among others (Hoang et al., 2022; Merimi et al., 2021). Indeed, several evidence showed that MSCs accelerate the tissue repair process occurring during wound healing, by migrating in the injured site and releasing of several factors involved both in tissue regeneration and reduction of the inflammatory state (Li et al., 2019). For these reasons, several clinical trials for MSC-based therapies have been registered worldwide (Moll et al., 2020). However, it is becoming increasingly accepted that the benefits of a stem cell-based therapy are mainly due to the combination of bioactive molecules that MSCs produce and release both in the growth medium and microenvironment (Lozito and Tuan, 2011; Bhang et al., 2014; Chen et al., 2018; Chudickova et al., 2019). In the strictest sense, the broad spectrum of these factors released in the liquid phase of the MSC culture environment is referred to as the conditioned medium (Dowling and Clynes, 2011; Joseph et al., 2020).

From a therapeutic standpoint, a cell-free approach based on conditioned medium provides several benefits over stem cell-based treatments, essentially because it does not result in any adverse events associated with MSCs administration such as rejection, malignant transformation, risk of thrombosis and/or calcification, thereby significantly improving the patient safety profile (Gunawardena et al., 2019). Not to mention that conditioned media can be obtained, transported and stored more easily than MSCs, without any ethical constrains (Gunawardena et al., 2019; Zhao et al., 2021).

With this premise, optimization of MSCs manufacturing workflow is a critical step to make a copious, highly reproducible, and safe production of conditioned medium. Since the number of published papers focusing on conditioned medium production and characterization has increased exponentially over the last 20 years (Di Santo et al., 2009; Zhao et al., 2015; Kay et al., 2017; Aboutaleb et al., 2019; Saheli et al., 2020; Kuo et al., 2021), conditioned medium can be considered a promising pharmaceutical product in regenerative medicine, due to anti-apoptotic and anti-inflammatory effects, neurotrophic and neuroprotective activity, and wound healing and tissue repair properties (Chen et al., 2008; Vizoso et al., 2017).

Nonetheless, these and other studies revealed that quali-quantitative composition and biological performance of conditioned medium preparations are heavily affected by a too high degree of variability in terms of MSC sources, donors, cell expansion, cell passage number, conditioning period, cell culture medium, microenvironment cues, and conditioned medium purification processes (Clabaut et al., 2015; Lukomska et al., 2019). It follows that standardization of manufacturing methods and protocols is essential to the development of conditioned medium-based therapeutic devices. Media supplemented with FBS are widely used to provide a supportive environment for isolation and expansion of MSCs from various sources, despite notorious practical, clinical, and ethical concerns over FBS use, essentially due to the presence of undesirable toxins, pathogen agents and/or xenogeneic proteins (Shahdadfar et al., 2005; van der Valk and Gstraunthaler, 2017). For this reason, in order to develop a clinically suitable and hazardous risk-free protocol for the isolation of Wharton’s jelly-derived MSCs (WJ-MSCs) from umbilical cords, many reports showed the potential of human platelet lysate (hPL) in enhancing MSCs recovery and propagation (de Soure et al., 2017; Becherucci et al., 2018; Kandoi et al., 2018; Vennila et al., 2019; Liao et al., 2021).

Actually, umbilical cord is a rich source of MSCs. This anatomical structure can be easily obtained during childbirth, and because it is considered an anatomical waste, it can be reused for scientific research without any ethical controversy (Bordet et al., 2010). Importantly, the umbilical cord represents an immune-privileged compartment that protects the fetus from the insults of the adult environment (Troyer and Weiss, 2008; Deuse et al., 2011; Fong et al., 2011), and in this regard WJ-MSCs show unique hypoimmunogenicity and tolerogenic properties in comparison with other MSCs (La Rocca et al., 2009; La Rocca et al., 2013). Moreover, WJ-MSCs are primitive stem cells displaying a multiplicity of unique properties attributed to their higher proliferative rate coupled with low senescence and higher production of trophic factors (La Rocca et al., 2009). Last, but not least, they fully satisfy the set of standard criteria for MSCs recommended by the International Society of Cytotherapy (Dominici et al., 2006).

Here we provide an optimized protocol for conditioned medium preparation starting from enzymatic-free explant culturing of umbilical cord fragments. We efficiently isolated and propagated WJ-MSCs in culture medium supplemented with commercial hPL, and appraised their morphology, proliferation, immunophenotyping, and differentiation abilities. Worth mentioning, compositional analysis of conditioned medium samples obtained from different WJ-MSC populations at different time points of conditioning revealed no substantial differences in the capacity of cells from different donors to produce comparable amounts of paracrine factors, when they are isolated, grown and induced, under the standardized conditions described in our protocol.

## 2 Materials and methods

### 2.1 Isolation, culture expansion, and phenotypic analysis of WJ-MSCs from umbilical cord

Umbilical cords (*n* = 19) were collected during caesarian delivery after full-term births with written informed consent from mothers according with the tenets of the Declaration of Helsinki and with a protocol approved by the Ethical Committee at the Azienda Ospedaliera Ospedali Riuniti Villa Sofia-Cervello (approval case number 331, 08/11/2016). Following incubation for 1 h in a sterile solution of cold Hank’s balanced salt (EuroClone) containing 200 U/mL penicillin and 200 mg/mL streptomycin (PAA Laboratories), umbilical cords were processed within 12 h from partum and WJ-MSCs isolated by migration and attachment to uncoated plates, using an improved version of a previously reported protocol (Lo Iacono et al., 2018). Briefly, umbilical cord segments of 40 cm on average in length were cut into small pieces of about 1 cm, each sectioned longitudinally to expose the Wharton’s jelly matrix to the plastic surface of 6 well culture treated plates (CytoOne), and incubated in a humidified atmosphere containing 5% CO_2_ at 37°C, in the following complete growth medium: Dulbecco’s Modified Eagle’s medium (DMEM) low-glucose (Euroclone), 1x non-essential amino acids (MEM, 100x, Sigma), 200 μML-glutamine, 100 U/mL penicillin, and 100 mg/mL streptomycin (PAA Laboratories), supplemented either with 5% IsoCell GROWTH hPL (EuroClone) or 10% heat inactivated fetal bovine serum (FBS; ThermoFisher) in some selected experiments.

The medium was changed every 2 days, and after 14 days cord fragments were removed. Next, cells attached to the plastic surface were cultured until reaching the 80% confluence, washed with 1X PBS, collected by enzymatic treatment with 50 μL/cm^2^ of Accutase solution (Accutase gentle solution for cell detachment, Euroclone), centrifuged at 1,000 g for 5 min, and plated at a density of 4.000 cells/cm^2^ in culture treated flasks (passage 1). For subsequent passages, the attached cells were treated and plated in the same medium as for the isolation step. The resulting cells at each passage were aliquoted at approximately 1 × 10^6^ cells per vial and cryopreserved for later use at −150°C in liquid nitrogen in 90% FBS with 10% DMSO.

The freeze-dried IsoCell GROWTH (Euroclone) derived from human platelet rich plasma of 100 donors, which results in a reduction in terms of variability of the different batches, providing a standardized platelet concentration. In preliminary experiments, we tested different concentrations (2.5%, 5%, and 10%) of IsoCell GROWTH to evaluate the proliferation kinetics of the WJ-MSCs at different time points (data not shown), and decided to use a 5% concentration for the subsequent experiments.

Cell count and viability during passages were evaluated by Trypan blue dye exclusion in the Burker chamber under a light inverted microscope (Leica DM-IL). The population doubling time (PDT) was calculated as follows: PDT = t x log(2)/log(Nt)-log (N0), where *t* is the time for cell culture (unit: hour), *Nt* is the number of cells after the culture, and *N0* is the number of cells initially plated.

Flow cytometric analysis (Beckman Coulter FC-500) was performed to confirm the mesenchymal signature of isolated WJ-MSCs populations. The detached cells were washed twice with PBS containing 0.5% BSA (Sigma-Aldrich), and marked with the following anti-human conjugated antibodies: anti-CD90-PC5, anti-CD105-PC7, anti-HLA-DR-PE, anti-CD31-PE, and anti-CD34/CD45-ECD (all from Beckman Coulter), and anti-CD73-FITC (BD Biosciences). About 1 × 10^5^ cells were used for each staining, and the data were analyzed by FlowJo software v10.

### 2.2 Adipogenic and osteogenic differentiation

WJ-MSCs at fourth passage were plated in 6-well plates at a density of 4.000 cells/cm^2^ and cultured in complete medium until 60% confluence. Then, the medium was changed with specific induction medium. For adipogenic induction, medium consisted of complete medium containing 1 µM dexamethasone, 5 μg/mL insulin, 0.5 mM isobutylmethylxanthine, and 200 µM indomethacin, while for osteogenic induction, complete medium containing 50 μM ascorbate, 10 mM sodium β-glycerophosphate, 0.01 µM dexamethasone, and 100 U/mL penicillin/streptomycin was used (all reagents from Sigma-Aldrich). After 3 weeks of induction, the cells were fixed with 4% formaldehyde and stained using 2% oil-red O or 1% alizarin-red S solution for the adipogenic and osteogenic differentiation, respectively. Images were collected using a Leica DM-IL microscope.

### 2.3 Conditioned medium preparation

WJ-MSCs at fourth passage, derived from four distinct umbilical cords, were cultured at a density of 4.000 cells/cm^2^ in T75 flasks in DMEM supplemented with 5% hPL to 80% confluence (2.5 ± 0.24 × 10^6^ cells/flask). Then, the attached cells were gently washed twice with 1X PBS, the complete medium was replaced with 9 mL of hPL-free DMEM, and the resulting conditioned medium was harvested following 24, 48, 72, and 96 h of incubation. At each time point, the collected medium was centrifuged for 10 min at 3,000 rpm, and the supernatant was 0.2 μm filtered to remove cell debris. In selected experiments, conditioned medium was concentrated ∼50-fold by ultrafiltration using Amicon Ultra-15 centrifugal filter devices with 10 kDa nominal molecular weight limit (Millipore). To this purpose, 15 mL of conditioned medium was collected in Amicon Ultra-15 tubes and centrifuged at 4.000 g for 25 min. Total protein quantification was performed by Bradford assay according to manufacturer’s instruction (Pierce Bradford assay, ThermoFisher Scientific). Both non-concentrated and concentrated conditioned medium preparations were stored at −80°C until use.

### 2.4 Secreted factor profiling of conditioned medium

Cytokines, chemokines, growth and trophic factors contained in conditioned media from four randomly selected cords, named CM 1 to 4, were screened using the Quantibody Human Arrays (RayBiotech). Based on the multiplexed sandwich ELISA method, 39 angiogenesis- (QAH-ANG-1000–1) and 19 inflammation-related (QAH-INF-3-1) factors, as well as 21 factors overlapping the two previous groups, were screened simultaneously following the manufacturer’s instructions. Briefly, each array membrane containing immobilized capture antibodies for the factors was incubated overnight at 4°C with 100 μL of unconcentrated conditioned medium or different dilutions of the supplied standard solutions containing known amounts of each factor to be detected. Thereafter, the biotin-conjugated secondary antibody was added and incubated for 2 h at room temperature. Finally, by means of a biotin-streptavidin interaction, Cy3-conjugated streptavidin bound to the antibody-protein complex was detected by a laser scanner system (Raybiotech) and factor concentration was calculated based on linear regression standard curves by comparing the densities of individual spots (each antibody was arrayed in quadruplicate) using Q-Analyzer software (Raybiotech). The negative controls included in the arrays were used to calculate the lower limit of detection for each factor, which is indicated in Supplementary Table S1. Beyond this limit, a <1.5 fold increase or decrease in concentration for a given factor among conditioned medium samples was considered a irrelevant difference.

Following this screening, TGFβ1, HGF, TIMP-1 and PDGF-BB factors were randomly selected and individually determined by conventional ELISA (Raybiotech) in samples of conditioned medium collected after 48 h, to validate the quantification values obtained with the Quantibody platform. The limits of detection for TGFβ1, HGF and TIMP-1 in the ELISA assay were 18–4.000 pg/mL, 3–2.000 pg/mL, and 40–18.000 pg/mL, respectively.

### 2.5 Scratch-wound healing assay

WJ-MSCs were seeded into 6-well plates at a density of 4.000 cells/cm^2^ and cultured under standard conditions until reaching 70% confluence. Then, cells were scratched using a sterile 200 μL pipette tip, washed twice with PBS to remove detached cells, and incubated in the presence of fresh growth medium either alone or supplemented with 5% hPL as negative and positive control, respectively, or in the presence of growing concentrations (1X and 2X) of CM1 collected after a conditioning period of 48 h. CM1 1X and 2X solutions were obtained by diluting with hPL-free growth medium the concentrated CM1 preparation. The images of migrating cells were captured at 0, 9 and 24 h of continuous culture, using a Leica DM-IL inverted microscope. Results were analyzed with ImageJ software (https://imagej.nih.gov/ij/), and presented as the percentage of wound healing calculated as follows: [wound area (0 h) − wound area (9 or 24 h)]/wound area (0 h) × 100.

### 2.6 Migration and invasion assay

The ability of conditioned medium to attract WJ-MSCs was tested in a 24-Transwell plate system (Corning), consisting of communicating chambers separated by inserts with semi-permeable PET membranes with 8 μm pore diameter. In particular, 3 × 10^4^ WJ-MSCs of two different batches at fourth passage were seeded into the upper transwell chamber containing 600 μL of DMEM supplemented with 0.5% FBS, and allowed to migrate toward conditioned medium samples collected after a conditioning period of 48 h. In these experiments, 5% IsoCell GROWTH hPL was used as a positive attractant control. After 16 h of incubation at 37°C, 5% CO_2_ in a humidified atmosphere, the cells on the upper surface of the transwell membranes were removed with cotton swabs and the cells migrated on the lower surface of the upper chamber were counted after Trypan blue staining under a light inverted microscope (Leica DM-IL). The percentage of cells migrated through the insert over the initial plated cells was calculated, and the assay was repeated twice in triplicate using four distinct conditioned medium preparations.

### 2.7 Cell viability and cell proliferation assays

WJ-MSCs at fourth passage were cultured for 72 h in DMEM (as a negative control), or in DMEM containing 5% IsoCell GROWTH hPL (as a positive control), or in distinct conditioned medium preparations collected after 48 h. Then, the viability of cells and the number of apoptotic cells were evaluated by the Annexin V staining assay (Beckman Coulter). Cells were washed twice with ice-cold PBS, resuspended in 1X Annexin V-FITC reagent, and kept at room temperature for 15 min protected from light. After the incubation period, samples were analyzed immediately by flow cytometry.

For the cell proliferation assay, WJ-MSCs at third passage were plated in 6-well plate (3 × 10^5^ cells/well), in the presence of distinct conditioned medium preparations collected after a conditioning period of 48 h. The number of cells was counted by trypan blue staining after 72 h of culture.

## 3 Results

### 3.1 Isolation and culture expansion of WJ-MSCs

#### 3.1.1 hPL supplementation increases the recovery of WJ-MSCs from umbilical cord

**FIGURE 1:**
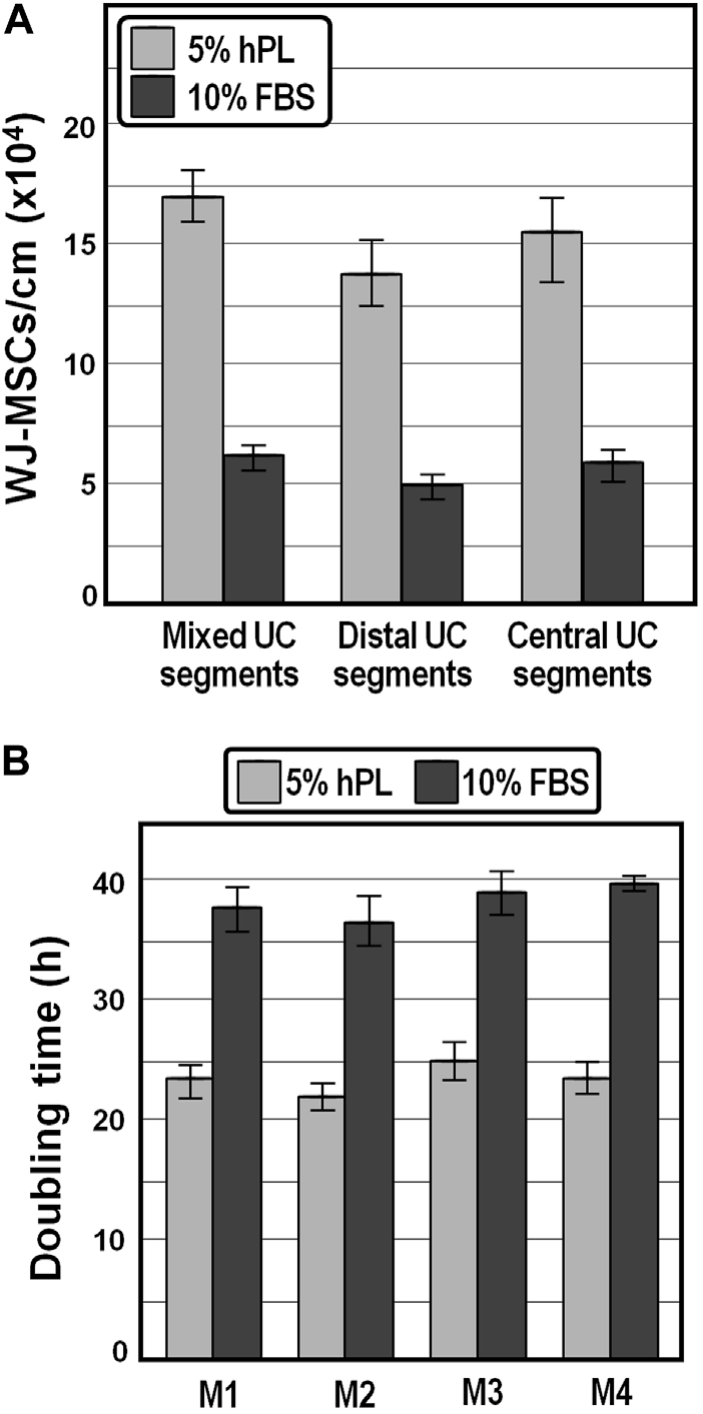
Yield and population doubling time of WJ-MSCs isolated from human umbilical cord. **(A)**, Average cell yields of WJ-MSCs obtained from mixed, distal and central sectors of distinct umbilical cords. **(B)**, Population doubling time of WJ-MSCs isolated from four distinct donors and grown for passages 1–6 in DMEM containing 5% hPL or 10% FBS.

As a first approach to examine the potential of hPL in enhancing WJ-MSCs recovery and propagation, umbilical cord segments collected after cesarean delivery were cut into small equal parts, each split longitudinally, and WJ-MSCs were isolated in the presence of medium containing 5% IsoCell GROWTH hPL or 10% FBS, respectively, as described in Materials and Methods. We observed that, compared to FBS, the number of WJ-MSCs released per cm of cord after 14 days of culture was increased by approximately 2.7-fold in the presence of hPL (Figure 1A), highlighting that hPL provides stronger chemoattractants that positively influenced the migration of WJ-MSCs. We also appraised that, although no statistically relevant differences in the average number of cells harvested from different (distal and central) parts within the same cord were detected (*p* > 0.75, one-way ANOVA), once again the yield was about 2.4-fold higher in the presence of hPL (Figure 1A).

#### 3.1.2 hPL supplementation significantly decreases the mean WJ-MSC population doubling time

We expanded the isolated WJ-MSCs for passages 1 to 6 and observed that the mean population doubling time was markedly lower (∼23.5 h on average) in the presence of 5% hPL compared with that of cells at the same generation cultivated in 10% FBS (∼38.2 h), possibly owing to a stronger effect of hPL on the proliferative capacity of WJ-MSCs (Figure 1B). Moreover, the average doubling time was comparable, although slightly shorter, with literature values reported for WJ-MSCs isolated by explant culture procedures (Fong et al., 2010; Majore et al., 2011; Venugopal et al., 2011).

#### 3.1.3 hPL supplementation allows maintenance of MSC key features

**FIGURE 2:**
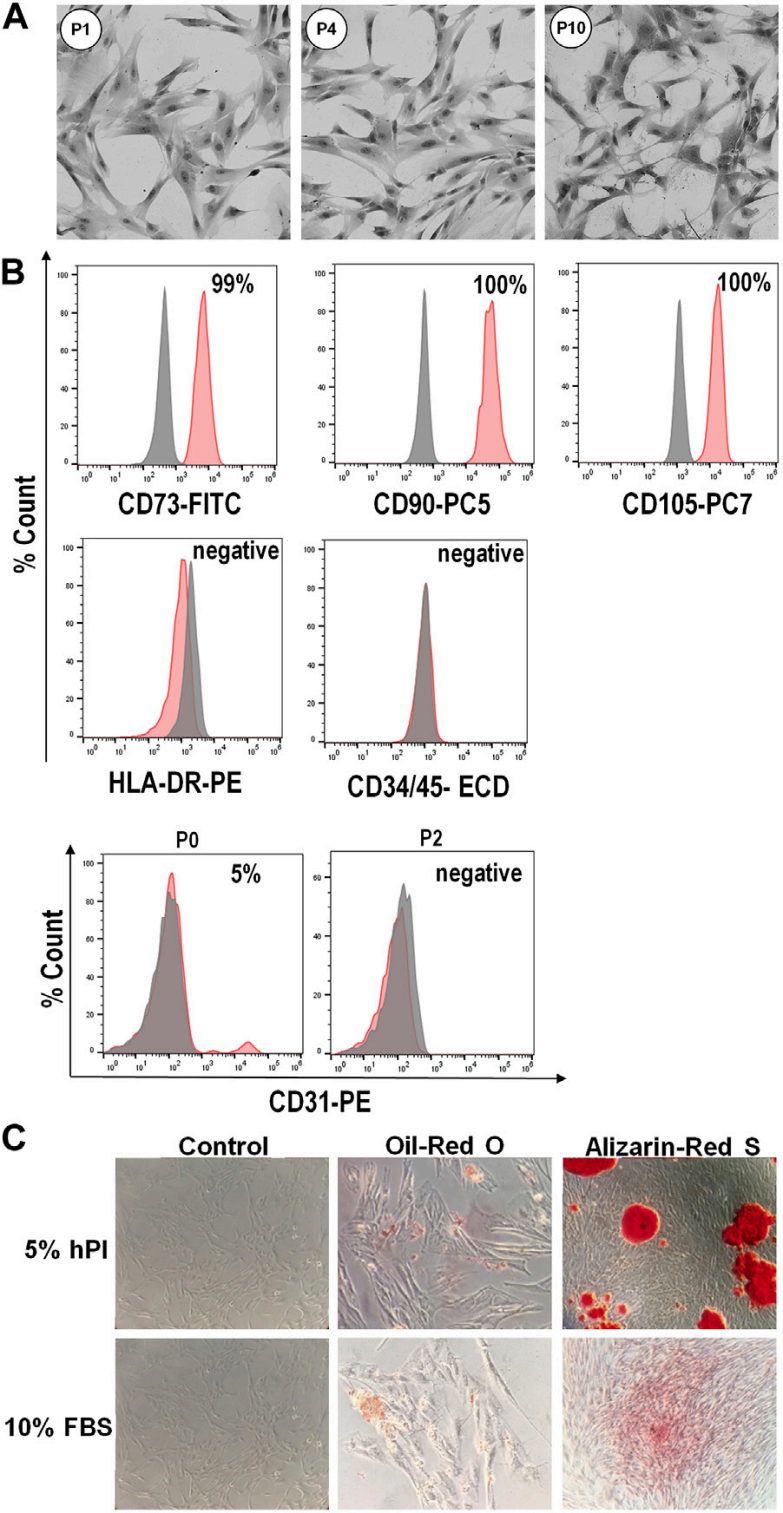
Phenotype and differentiation potential of WJ-MSCs isolated from umbilical cord. **(A)**, Representative images showing typical morphology of isolated WJ-MSCs at the indicated culture passages. **(B)**, Flow cytometry analysis of surface markers of WJ-MSCs at passage 10, showing positive expression (99%–100%) for the canonical mesenchymal cell markers CD73, CD90 and CD105, and negative expression (<2%) for HLA-DR, CD34, CD45, and CD31. Red color indicates the percentage of positive cells for different markers, while grey color indicates isotype match control; **(C)**, Staining for adipogenic and osteogenic differentiation. WJ-MSCs cultured for 3 weeks in adipogenic or osteogenic medium, as well as in control medium, were stained with oil-red O or alizarin-red S, respectively.

The WJ-MSCs isolated in the presence of hPL retained a typical fibroblastoid morphology for several subsequent passaging (Figure 2A). Flow cytometry analysis revealed that these cells, from passages 0 to 10, were highly positive for canonical mesenchymal cell surface markers such as CD73, CD90 and CD105, and negative for the expression of CD34 and CD45 hematopoietic markers (Figure 2B). Moreover, isolated WJ-MSCs did not express the major histocompatibility complex class II antigen HLA-DR (Figure 2B), which allows them to evade immune surveillance. We also observed that at passage 0, when primary cells move from cord pieces to culture medium containing either hPL or FBS, about 5%–15% of cells expressed the endothelial marker CD31 (Figure 2B), suggesting that these cells most probably came from the umbilical vein of the cord. Nevertheless, CD31 became no longer detectable since the first passage, confirming that these cells did not survive or propagate in these culture conditions (Figure 2B). Finally, cells isolated from different segments of the umbilical cord in the presence of hPL had the potential to differentiate into adipocytes and osteoblasts, as revealed by oil-red O and alizarin-red staining, respectively indicating accumulation of lipid vacuoles and the presence of calcium deposits (Figure 2C).

Overall, these results confirmed that a uniform population of WJ-MSCs was successfully isolated from human umbilical cord and efficiently propagated by using a culture medium containing hPL as an alternative to FBS.

### 3.2 Production and characterization of conditioned medium from WJ-MSCs

#### 3.2.1 Harvesting of conditioned medium

WJ-MSCs isolated from 19 distinct umbilical cords, and characterized by immunophenotyping and differentiation ability, were used to prepare conditioned medium as described in Materials and Methods. Briefly, WJ-MSCs at fourth passage were initially cultured at a density of 4.000 cells/cm^2^ in DMEM supplemented with 5% hPL to 80% confluence. Then, the complete medium was replaced with hPL-free DMEM, and the resulting conditioned medium was harvested and eventually concentrated by ultrafiltration after 24, 48, 72, and 96 h of incubation.

**FIGURE 3:**
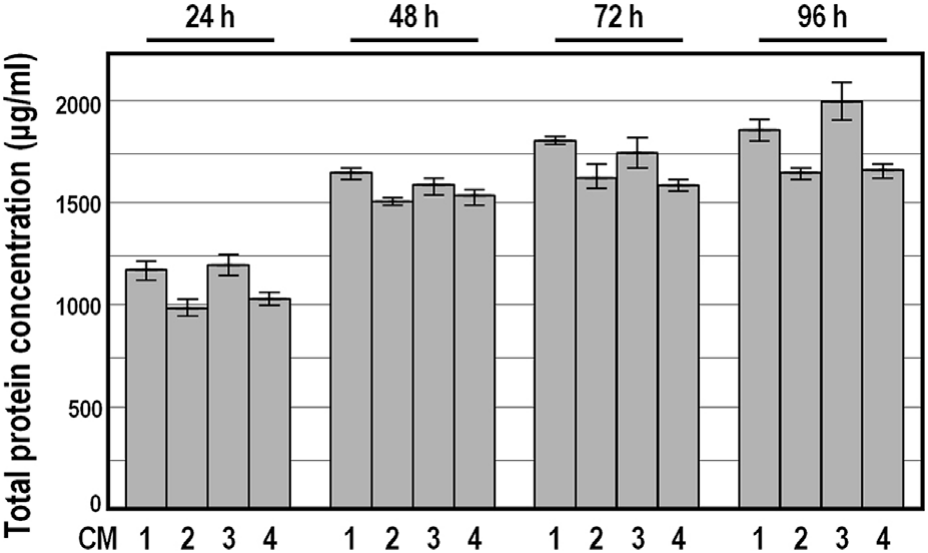
Total protein concentration in conditioned medium preparations derived from four distinct donors. Data are expressed in μg/mL as means ± SD, measured by Bradford assay. Protein concentration was below the detection limit in DMEM.

Because Bradford assay revealed a broadly similar total protein amount in these conditioned medium preparations, and ELISA assays confirmed the absence of PDGF-BB and the presence of congruent abundances of TGFb1, HGF and TIMP-1 factors among the 19 CM preparation series (see below), we focused on four randomly selected conditioned medium preparations, named CM 1 to 4, for further characterization. As expected, the total protein amount determined by Bradford assay in concentrated conditioned medium samples was considerably higher compared with DMEM (Figure 3). Moreover, the kinetics of protein secretion over conditioning time showed a comparable donor-independent increase in the total protein amount, with the smallest difference at 48 h of incubation (Figure 3).

#### 3.2.2 Compositional analysis of conditioned medium

To better clarify the secretory profile of WJ-MSCs during conditioning, conditioned medium samples were screened using the Quantibody platform to simultaneously quantify a set of 79 factors, of which 39 related to angiogenesis, 19 to inflammation, and 21 overlapped the two previous groups. As expected, most of the proinflammatory and/or immunomodulatory cytokines (n = 51/79), including IL-1α, IL-1β, IL-4, IL-5, IL-10, IL-12p40, IL-12p70, IL-13, IL-16, IL-17A, IFN-γ, TNF-α and -β, were either undetected or extremely close to the lower limit of detection (LOD) in all samples and could not be analyzed (Supplementary Table S1). Signals above the LOD were observed for 28 out of 79 factors assayed, conjointly exhibiting a strictly similar accumulation trend over the four time points examined, with a notable increase in concentration up to 48 h of incubation (Supplementary Table S1). Importantly, at this conditioning time point there were no obvious differences across the four conditioned medium samples in the abundance of almost all of these factors (n = 26/28), including those involved in the wound healing cascade (HGF, VEGF-A/-D, IGF-1, and TGFβ1) (Hoang et al., 2020), as well as those involved in extracellular matrix remodeling (MMP-1, TIMP-1, and uPAR) (Wang et al., 2017), and factors commonly found in conditioned medium from various sources (GCSF, LIF, CCL-2, -5 and -7, CXCL-1/2/3 and −5, ICAM-1, IL-6, -8 and -11) (Hsiao et al., 2012). The only discrepancy pertained the ANGPTL4 and IL-11 factors, both significantly underrepresented in CM1 (Supplementary Table S1). In particular, compared to CM1, the average concentration of ANGPTL4 was about 2.8-fold higher in CM2, 2-fold higher in CM3, and 2.2-fold higher in CM4, while the average concentration of IL-11 was about 1.9-fold higher in CM2, 8.1-fold higher in CM3 and 7.5-fold higher in CM4 (Supplementary Table S1). Since profiling of secreted factors highlighted minimal inter-individual variability in conditioned medium at 48 h, we focused on this conditioning time point conditioned medium for subsequent experiments.

**FIGURE 4:**
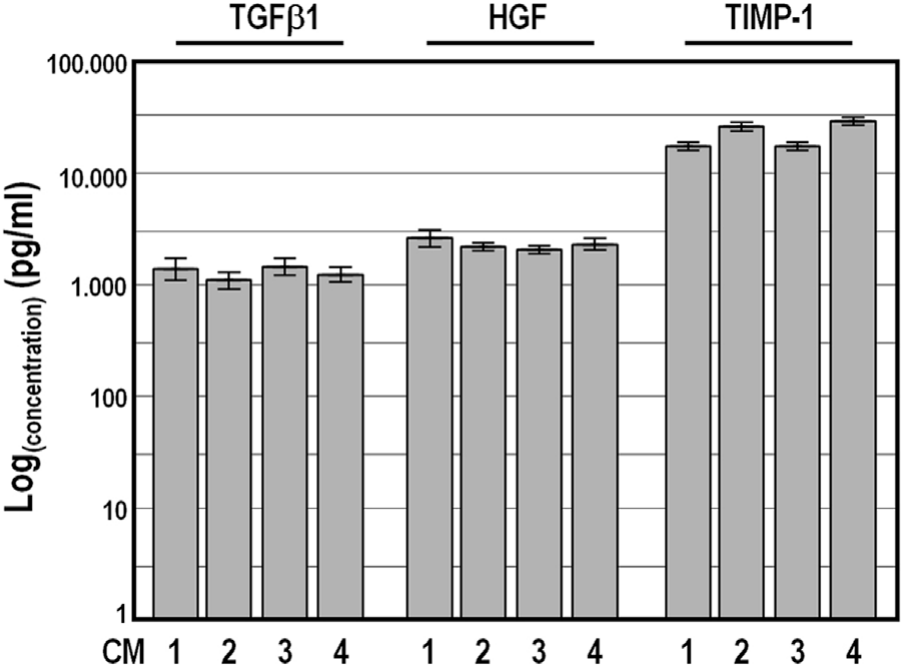
Levels of TGFβ1, HGF, and TIMP-1 factors determined by conventional ELISA analysis in four distinct conditioned medium samples harvested after a conditioning period of 48 h. Concentration values ±SD are given on logarithmic scale.

To validate the reliability of the Quantibody outcomes, we chose four factors, namely, TGFβ1, HGF, TIMP-1 and PDGF-BB, and quantified their amount by conventional single target ELISA in the four conditioned medium samples collected at 48 h. As expected, PDGF-BB abundance was below the ELISA detection limit, while the values obtained for TGFβ1, HGF and TIMP-1 were strictly congruent with those from the Quantibody assays, confirming the high concordance between the two detection methods (Figure 4).

Altogether, these results strongly suggest that there are no substantial differences in the capacity of WJ-MSCs from different donors to produce paracrine factors when they are isolated, grown and induced, under the standardized conditions described in our protocol.

#### 3.2.3 Chemoattractant, cytoprotective, and anti-mitogenic properties of conditioned medium

**FIGURE 5:**
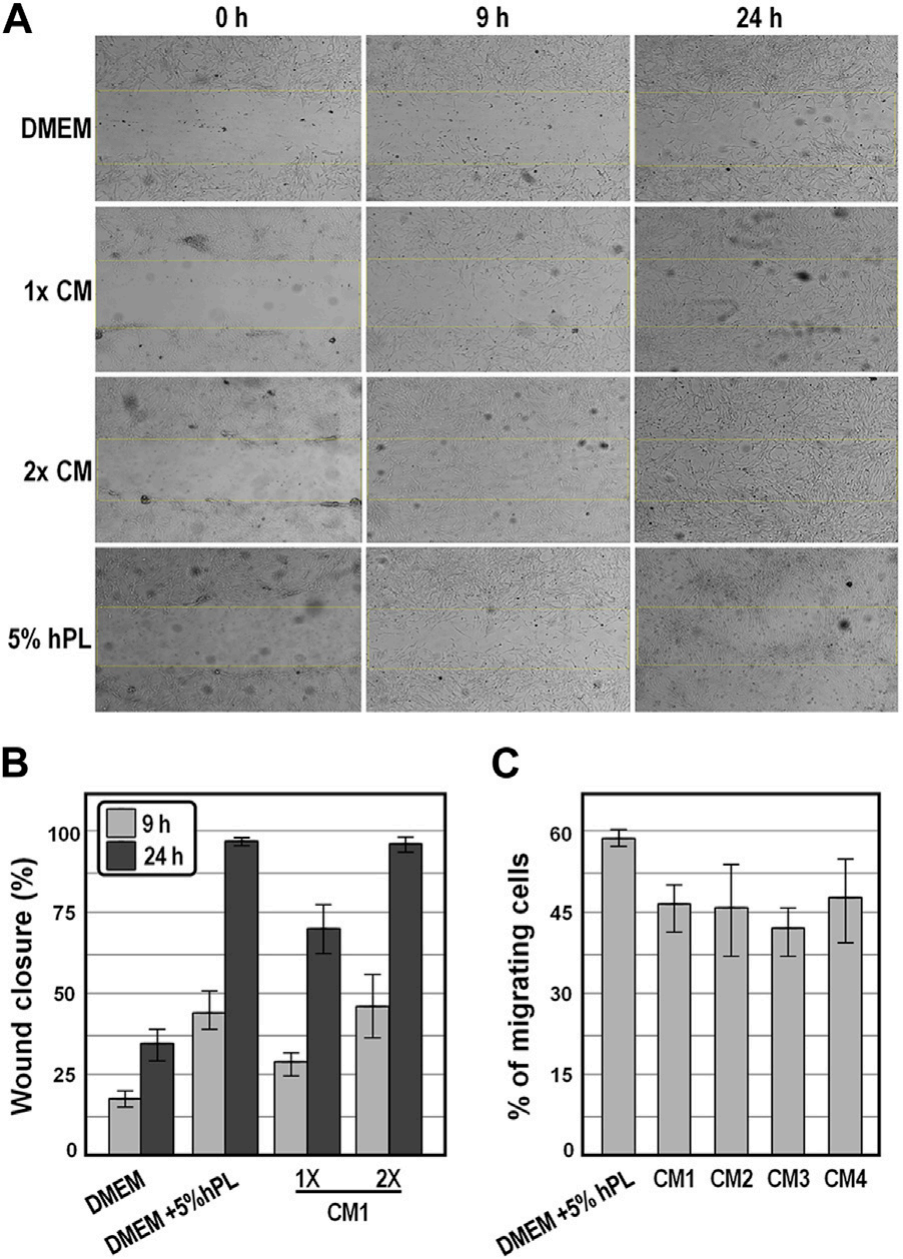
Analysis of the motility behavior and invasiveness of WJ-MSC exposed to conditioned medium. **(A)**, Representative bright-field images of the scratch-wound healing assay of WJ-MSCs exposed to the indicate conditions at the indicated time points. **(B)**, Graph showing the percentages of wound healing from the scratch-wound healing assay. **(C)**, Graph showing the percentage of migrated cells from the transwell migration assay after 16 h, in the indicated experimental groups. Values are means ± SD.

We next determined the chemoattractant properties of conditioned medium collected at 48 h on WJ-MSCs by scratch wound healing assay and by transwell migration and invasion assay. We observed that, in the presence of CM1, the wound was more effectively healed over 24 h as compared to the control group of cells exposed to DMEM (Figures 5A,B), and that comparable wound healing was obtained in the presence of either 2x CM1 or DMEM supplemented with 5% hPL (Figures 5A,B), suggesting that cell migration was enhanced in the presence of conditioned medium. In strict accordance, in the migration assay, comparable numbers of WJ-MSCs occupying the lower side of the transwell membrane were observed either in the presence of distinct preparations of conditioned medium or DMEM supplemented with 5% hPL (Figure 5C). These findings indicate that conditioned medium enhanced the motility behavior and invasiveness of WJ-MSCs *in vitro*.

**FIGURE 6:**
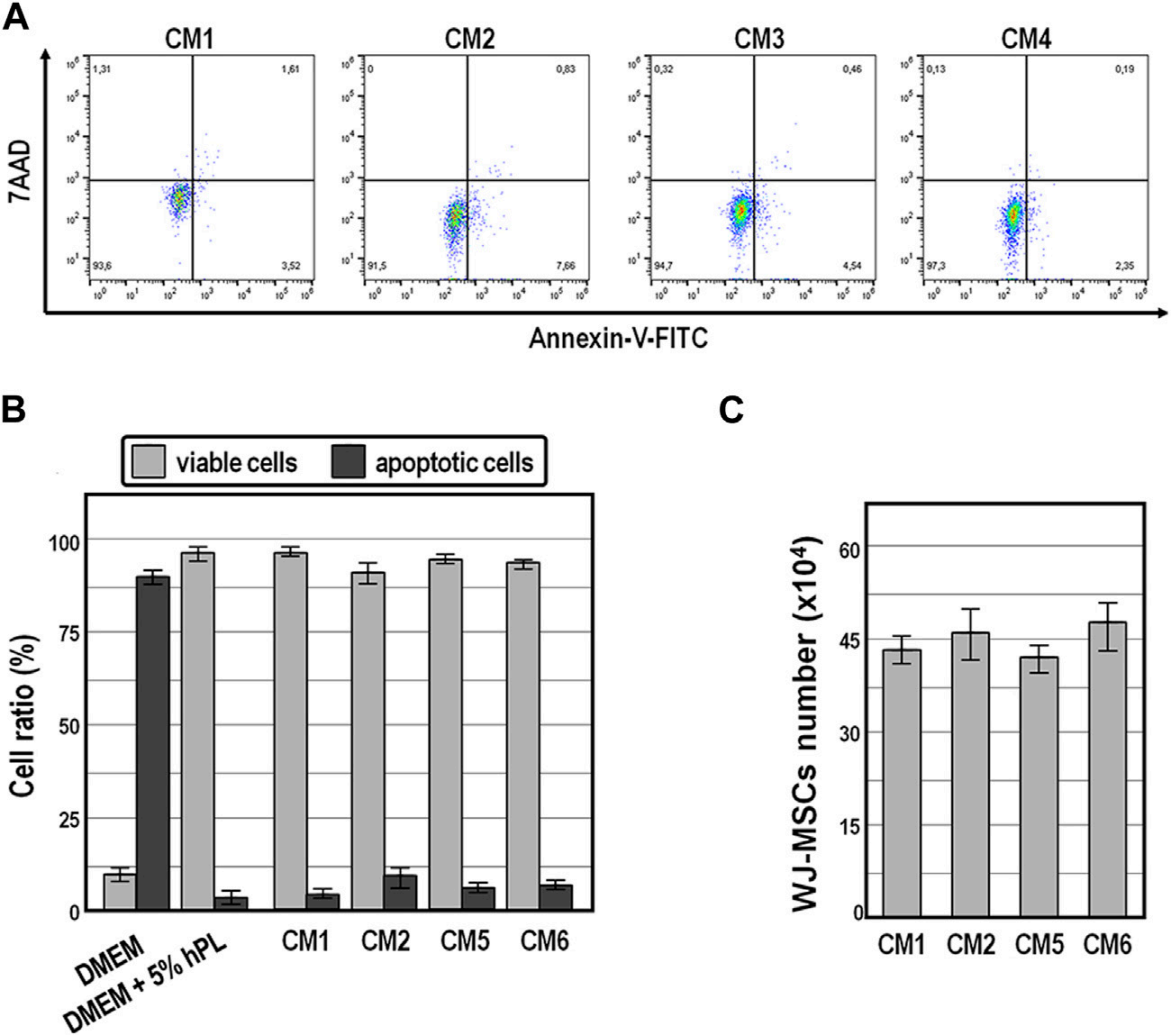
Cell viability and population size of WJ-MSCs exposed to conditioned medium. **(A,B)**, Cell viability and apoptosis evaluation by AnnexinV staining and cytometry analysis of WJ-MSCs at third passage cultured for 72 h in the indicated conditions. **(C)**, Population size of WJ-MSCs cultured for 72 h in the presence of the indicated conditioned medium preparations. Values are means ± SD.

Finally, annexin V-FITC staining assay revealed that while exposure of WJ-MSCs to ordinary DMEM induced apoptosis in the vast majority of cells (89% ± 7%) within 72 h (Figures 6A,B), exposure for the same time period to the four distinct conditioned medium preparations offered cytoprotective properties, as indicated by an average of 91.5%–97.3% of viable cells (Figures 6A,B).

Also noteworthy is the fact that the population size of WJ-MSCs cultured for up to 72 h did not change significantly in the presence of those conditioned medium preparations (Figure 6C), suggesting that conditioned medium exerted anti-mitogenic activity in WJ-MSCs.

## 4 Discussion

In this study, we optimized procedures aimed to determine an efficient means to extract and propagate WJ-MSCs from umbilical cord, and to prepare conditioned medium samples with homogeneous composition to fulfil therapeutic application in the field of protective and regenerative medicine.

Although FBS-supplemented media are commonly used for MSC expansion, FBS is a complex mixture of xenogeneic factors with high lot-to-lot variability and risk of pathogen contamination, which hampers translation to clinical for both expanded MSCs and their derived products (Shahdadfar et al., 2005; van der Valk and Gstraunthaler, 2017; Mushahary et al., 2018). Here we confirm that the use of hPL-supplemented medium not only overcomes the drawbacks associated with FBS, but also improve both the yield and expansion efficiency of WJ-MSCs, maintaining their differentiation and secretory potential.

One of the main challenges in cell-free therapy with conditioned medium preparations is to obtain an easily expandable cell system, which might provide a scale-up framework suitable for the production of industrial amounts of conditioned medium with predictable composition (Das et al., 2019). Importantly, by using our optimized protocol, approximately six million WJ-MSCs can be harvested from a single umbilical cord of about 40 cm in length. The serial replating of these cells until the fourth passage, at the concentration of 4.000 cells/cm^2^ for each passage, theoretically allows the preparation of about 120 L of conditioned medium containing a great variety of biomolecules such as cytokines, interleukins, growth and trophic factors.

It is worth mentioning that, under these conditions, the compositional profile of distinct conditioned medium preparations does not depend on donor individuality, as very similar accumulation trend and abundance of the same set of factors is detected from distinct umbilical cords. Among those factors, we detected angiogenic molecules (HGF, TGF-β1, VEGF-A and -D) and chemokines involved in the neutrophil, macrophages, and lymphocytes recruitment (IL-8, CCL-2 and 5, CXCL-1 and -8). The only discrepancy in terms of concentration among the four conditioned medium preparations analyzed pertained the multifunctional ANGPTL4 and IL-11 cytokines, both known to play disparate roles in healthy cells and in various pathologies. In particular, ANGPTL4 is involved in different aspects of lipid metabolism and vascular function and dysfunction (Fernández-Hernando and Suárez, 2020), while IL-11 can act as an anti-inflammatory cytokine (Trepicchio et al., 1996) and thrombopoietic factor (Schlerman et al., 1996), and it has been shown to play a role in B- and T-cell differentiation and antibody production (Anderson et al., 1992). Other studies implicated IL-11 with the onset and progression of fibrotic diseases (Schafer et al., 2017; Widjaja et al., 2019), autoimmune diseases (Hermann et al., 1998; Figueiredo et al., 2014; Adami et al., 2021), Alzheimer’s disease (Galimberti et al., 2008; Pellicanò et al., 2009) and various types of cancer diseases (Lay et al., 2012; Putoczki et al., 2013; Liang et al., 2019). Whether the elevation of circulating ANGPTL4 and IL-11 is pathogenic or a natural response to restore homeostasis is not clear for many diseases. Whatever is the case, most probably the observed differences in ANGPTL4 and IL-11 abundance in conditioned media derived from distinct donors reflect dissimilar epigenetic state at the *angptl4* and *il-11* loci. In fact, it is widely accepted that the epigenome can act as the link between environmental cues, both external and internal, to the organism and phenotype by converting the environmental stimuli to phenotypic responses through changes of gene transcription outcomes (Di Caro et al., 2007; Cavalieri et al., 2009; Cavalieri et al., 2013; Cavalieri, 2020a; Cavalieri, 2020b; Cavalieri, 2021; La Rocca et al., 2021). Accordingly, individual differences in *il-11* gene expression have been related to distinct epigenetic landscapes aroused after antidepressant treatment (Powell et al., 2013). Similarly, positive correlation among increased chromatin enhancer activity, gene transcription and DNA hypomethylation has been described for the *angptl4* gene in the atherosclerotic chromatin (Ehrlich et al., 2019).

In a derivative study (Reina et al., 2023), we assessed the biological effects *in vivo* elicited by exposure to the conditioned medium preparations described here. Intriguingly, by using the zebrafish model we found that conditioned medium treatment triggers multiple favourable outcomes *in vivo*, including antioxidant, anti-apoptotic and pro-regenerative effects, impinging on specific marker gene expression. Furthermore, these findings confirm that the observed effects do not depend on donor individuality of conditioned medium preparations.

As mentioned, conditioned media are complex mixtures of active biological factors, whose composition and concentration are strictly dependent from donor, cell source, culture medium, and conditioning time, among others. The combination of these facets heavily hinders the implementation of robust and reliable potency tests, which are absolutely required for defining a biopharmaceutical product. According to the FDA, EMA, and International Conference Harmonization (Teasdale et al., 2017), potency is defined as a quantitative measure correlated with a relevant biological activity, and a potency test should show a specific biological effect strictly related with a clinical response. The absence of potency tests for conditioned medium-based biopharmaceuticals also limits their classification within the regulatory framework. In fact, based on the opinion of the experts of the field, conditioned medium-based products should be allocated to a novel group of products placed at the intersection of the Biomedical Drugs and Advanced Therapies Medicinal Products categories. Therapeutically active substances falling within this group are not yet approved drugs, and they are referred to as Investigational Medicinal Products in Europe and Investigational New Drugs in United States. From a regulatory perspective, in the absence of a clear classification and regulatory framework by European Medicines Agency and Food and Drug Administration for this category of biopharmaceuticals, cell-derived conditioned media actually fall into a regulatory gap. In this context, our work towards the optimization of procedures for conditioned medium production and characterization both *in vitro* and *in vivo* will certainly help to define specific regulatory laws.

In conclusion, our optimized procedure allowed successful isolation of WJ-MSCs from umbilical cords and consequent obtaining of standardized conditioned medium preparations. Isolated cells displayed the ability to self-renew, expressed stem cell marker genes, maintained their differentiation potential, and were able to secrete a great variety of biomolecules with very low donor-specific variability. Moreover, all of the four conditioned medium preparations displayed broadly similar abundances of factors involved in wound healing, extracellular matrix remodelling, and immunomodulation processes. Based on these evidence, we conclude that our procedure will be helpful for the development of a cell-free based product with promising therapeutic potential in the field of regenerative medicine.

## Data Availability

The original contributions presented in the study are included in the article/Supplementary Material, further inquiries can be directed to the corresponding author.
