# Supplementary material for: An optimized procedure for preparation of conditioned medium from Wharton’s jelly mesenchymal stromal cells isolated from umbilical cord
Source: Front Mol Biosci. 2023 Oct 2;10:1273814. doi: 10.3389/fmolb.2023.1273814 (PMC10580810; doi:10.3389/fmolb.2023.1273814)
Supplement: Supplementary file 1 [file Table1.docx]

**Supplementary Table S1. Angiogenesis and inflammation-related factor quantification by Quantibody Human Arrays. Values are indicated in pg/ml, and asterisks indicate values below the LOD.**

|  |  |  | 24 h |  |  |  | 48 h |  |  |  | 72 h |  |  |  | 96 h |  | LOD |
| --- | --- | --- | --- | --- | --- | --- | --- | --- | --- | --- | --- | --- | --- | --- | --- | --- | --- |
|  | **CM1** | **CM2** | **CM3** | **CM4** | **CM1** | **CM2** | **CM3** | **CM4** | **CM1** | **CM2** | **CM3** | **CM4** | **CM1** | **CM2** | **CM3** | **CM4** |  |
| Activin A | * | * | * | * | 365 ±75 | 470 ±90 | 415 ±70 | 354 ±11 | 22853 ±2803 | 21718 ±2504 | 28678 ±3233 | 21173 ±2030 | 29441 ±2509 | 28064 ±1449 | 33793 ±2740 | 25434 ±3071 | 137 |
| AgRP | * | * | * | * | * | * | * | * | * | * | * | * | * | * | * | * | 6,75 |
| ANG | 9270 ±356 | 9474 ±644 | 6156 ±1434 | 10217 ±179 | 8980 ±570 | 11435 ±818 | 8734 ±958 | 12523 ±604 | 13792 ±1381 | 14748 ±1968 | 10262 ±705 | 13092 ±1218 | 10697 ±884 | 11576 ±2384 | 6277 ±450 | 9545 ±1316 | 370 |
| ANGPT1 | 321 ±27 | 290 ±36 | 311 ±29 | 167 ±9 | 4237 ±85 | 2900 ±175 | 3508 ±103 | 2823 ±133 | 4999 ±122 | 4570 ±239 | 4888 ±197 | 3877 ±519 | 3015 ±102 | 2413 ±712 | 3337 ±90 | 2005 ±647 | 67,5 |
| ANGPT2 | * | * | * | * | * | * | * | * | * | * | * | * | * | * | * | * | 10,8 |
| ANGIO | 150 ±36 | 150 ±38 | 122 ±24 | 144 ±19 | 1720 ±270 | 1499 ±338 | 1522 ±89 | 1650 ±221 | 1002 ±84 | 1137 ±222 | 1099 ±130 | 1121 ±66 | 917 ±97 | 801 ±98 | 1020 ±57 | 1214 ±164 | 27 |
| ANGPTL4 | 1886 ±111 | 3981 ±564 | 1654 ±117 | 1982 ±190 | 1880 ±601 | 5280 ±839 | 3803 ±532 | 4106 ±683 | 3312 ±455 | 8372 ±674 | 6348 ±463 | 10243 ±1136 | 4865 ±414 | 8557 ±185 | 2389 ±386 | 7651 ±735 | 270 |
| bFGF | * | * | * | * | * | * | * | * | * | * | * | * | * | * | * | * | 13,5 |
| CCL1 | * | * | * | * | * | * | * | * | * | * | * | * | * | * | * | * | 5,4 |
| CCL2 | 1901 ±436 | 1337 ±126 | 1709 ±427 | 1589 ±219 | 2343 ±202 | 2661 ±426 | 3033 ±377 | 2189 ±283 | 2296 ±203 | 2491 ±233 | 1903 ±256 | 1825 ±255 | 2040 ±366 | 2915 ±270 | 2160 ±620 | 2504 ±383 | 2,7 |
| CCL3 | * | * | * | * | * | * | * | * | * | * | * | * | * | * | * | * | 6,75 |
| CCL4 | * | * | * | * | * | * | * | * | * | * | * | * | * | * | * | * | 0,67 |
| CCL5 | 8860 ±790 | 9299 ±1496 | 8822 ±750 | 5423 ±764 | 7005 ±372 | 9070 ±1328 | 9980 ±396 | 9709 ±323 | 9111 ±720 | 8550 ±1209 | 7005 ±1100 | 6466 ±963 | 4663 ±1096 | 3236 ±148 | 5532 ±928 | 6201 ±829 | 27 |
| CCL7 | 750 ±89 | 998 ±160 | 1124 ±229 | 803 ±39 | 3550 ±100 | 5080 ±480 | 4880 ±289 | 4190 ±63 | 3468 ±790 | 5175 ±130 | 5320 ±255 | 5129 ±164 | 2027 ±10 | 1826 ±448 | 3500 ±98 | 3538 ±101 | 5,4 |
| CCL8 | * | * | * | * | * | * | * | * | * | * | * | * | * | * | * | * | 1,35 |
| CCL11 | 76 ±3 | 83 ±13 | 95 ±15 | 71 ±2 | 56 ±6 | 61 ±8 | 79 ±5 | 62 ±6 | 622 ±194 | 380 ±29 | 740 ±194 | 348 ±13 | 1004 ±185 | 714 ±116 | 1097 ±161 | 637 ±148 | 2,7 |
| CCL13 | * | * | * | * | * | * | * | * | * | * | * | * | * | * | * | * | 13,5 |
| CCL15 | * | * | * | * | * | * | * | * | * | * | * | * | * | * | * | * | 6,75 |
| CCL24 | * | * | * | * | * | * | * | * | * | * | * | * | * | * | * | * | 0,67 |
| CD31 | * | * | * | * | * | * | * | * | * | * | * | * | * | * | * | * | 27 |
| CXCL1/2/3 | 185 ±39 | 129 ±30 | 139 ±25 | 172 ±29 | 205 ±19 | 304 ±9 | 229 ±20 | 294 ±51 | 196 ±11 | 268 ±34 | 344 ±90 | 325 ±52 | 154 ±33 | 248 ±40 | 326 ±87 | 236 ±35 | 5,4 |
| CXCL5 | 310 ±18 | 196 ±51 | 207 ±23 | 170 ±30 | 2210 ±80 | 2208 ±159 | 3314 ±104 | 3190 ±209 | 3040 ±287 | 3814 ±832 | 5764 ±268 | 4620 ±1128 | 5979 ±290 | 5882 ±303 | 7637 ±1326 | 5848 ±495 | 13,5 |
| CXCL9 | * | * | * | * | * | * | * | * | * | * | * | * | * | * | * | * | 27 |
| CXCL11 | * | * | * | * | * | * | * | * | * | * | * | * | * | * | * | * | 2,7 |
| CXCL13 | * | * | * | * | * | * | * | * | * | * | * | * | * | * | * | * | 0,67 |
| CXCL16 | * | * | * | * | * | * | * | * | * | * | * | * | * | * | * | * | 2,7 |
| EGF | * | * | * | * | * | * | * | * | * | * | * | * | * | * | * | * | 0,07 |
| FGF-4 | * | * | * | * | * | * | * | * | * | * | * | * | * | * | * | * | 135 |
| FST | 640 ±18 | 910 ±151 | 615 ±12 | 821 ±71 | 1041 ±59 | 1135 ±99 | 1555 ±75 | 1311 ±91 | 379 ±98 | 547 ±49 | 562 ±25 | 580 ±125 | 297 ±30 | 376 ±67 | 377 ±45 | 545 ±177 | 135 |
| GCSF | 502 ±29 | 611 ±43 | 455 ±81 | 433 ±63 | 4709 ±522 | 4972 ±108 | 4607 ±109 | 5002 ±961 | 5847 ±302 | 6809 ±41 | 6228 ±191 | 7406 ±327 | 2926 ±299 | 2400 ±266 | 3819 ±302 | 3806 ±114 | 27 |
| GM-CSF | * | * | * | * | * | * | * | * | * | * | * | * | * | * | * | * | 2,7 |
| HB-EGF | * | * | * | * | * | * | * | * | * | * | * | * | * | * | * | * | 5,4 |
| HGF | 1534 ±174 | 1228 ±94 | 1976 ±187 | 1478 ±98 | 2490 ±180 | 2294 ±197 | 1944 ±46 | 2738 ±159 | 2441 ±689 | 2661 ±128 | 2209 ±485 | 3499 ±152 | 2532 ±172 | 2178 ±388 | 2562 ±154 | 3058 ±165 | 5,4 |
| ICAM-1 | 3891 ±437 | 3849 ±518 | 1804 ±406 | 1939 ±199 | 3678 ±181 | 2923 ±175 | 2979 ±215 | 4343 ±199 | 3579 ±362 | 4308 ±253 | 6256 ±331 | 5800 ±323 | 1865 ±191 | 1133 ±182 | 3536 ±399 | 1943 ±120 | 67,5 |
| IFN-γ | * | * | * | * | * | * | * | * | * | * | * | * | * | * | * | * | 2,7 |
| IGF-1 | 814 ±145 | 1162 ±99 | 1171 ±480 | 1079 ±218 | 1348 ±306 | 1501 ±387 | 1166 ±143 | 1105 ±293 | 872 ±87 | 1090 ±291 | 802 ±151 | 916 ±24 | 756 ±154 | 432 ±130 | 809 ±102 | 521 ±93 | 67,5 |
| IL-1α | * | * | * | * | * | * | * | * | * | * | * | * | * | * | * | * | 2,7 |
| IL-1β | * | * | * | * | * | * | * | * | * | * | * | * | * | * | * | * | 1,35 |
| IL-1ra | * | * | * | * | * | * | * | * | * | * | * | * | * | * | * | * | 0,67 |
| IL-2 | * | * | * | * | * | * | * | * | * | * | * | * | * | * | * | * | 5,4 |
| IL-4 | * | * | * | * | * | * | * | * | * | * | * | * | * | * | * | * | 1,35 |
| IL-5 | * | * | * | * | * | * | * | * | * | * | * | * | * | * | * | * | 2,7 |
| IL-6 | 1010 ±670 | 392 ±35 | 466 ±10 | 378 ±21 | 1407 ±301 | 1583 ±438 | 1976 ±44 | 1751 ±50 | 2146 ±163 | 1087 ±74 | 2297 ±102 | 1920 ±123 | 1047 ±127 | 1374 ±86 | 2480 ±106 | 1983 ±56 | 2,7 |
| IL-6R | * | * | * | * | * | * | * | * | * | * | * | * | * | * | * | * | 6,75 |
| IL-7 | * | * | * | * | * | * | * | * | * | * | * | * | * | * | * | * | 2,7 |
| IL-8 | 306 ±52 | 265 ±21 | 203 ±29 | 169 ±23 | 321 ±25 | 280 ±45 | 407 ±96 | 367 ±55 | 325 ±95 | 355 ±26 | 325 ±39 | 458 ±36 | 294 ±24 | 557 ±102 | 498 ±69 | 530 ±124 | 1,35 |
| IL-10 | * | * | * | * | * | * | * | * | * | * | * | * | * | * | * | * | 1,35 |
| IL-11 | 216 ±18 | 127 ±8 | 120 ±13 | 202 ±47 | 120 ±19 | 229 ±15 | 972 ±187 | 895 ±184 | 401 ±41 | 529 ±124 | 2627 ±688 | 2951 ±596 | 1431 ±277 | 1119 ±160 | 3430 ±426 | 3090 ±865 | 13,5 |
| IL-12 p40 | * | * | * | * | * | * | * | * | * | * | * | * | * | * | * | * | 13,5 |
| IL-12 p70 | * | * | * | * | * | * | * | * | * | * | * | * | * | * | * | * | 0,67 |
| IL-13 | * | * | * | * | * | * | * | * | * | * | * | * | * | * | * | * | 0,67 |
| IL-15 | * | * | * | * | * | * | * | * | * | * | * | * | * | * | * | * | 2,7 |
| IL-16 | * | * | * | * | * | * | * | * | * | * | * | * | * | * | * | * | 3,37 |
| IL-17A | * | * | * | * | * | * | * | * | * | * | * | * | * | * | * | * | 5,4 |
| IP-10 | * | * | * | * | * | * | * | * | * | * | * | * | * | * | * | * | 2,7 |
| Leptin | * | * | * | * | * | * | * | * | * | * | * | * | * | * | * | * | 54 |
| LIF | 95 ±29 | 102 ±40 | 49 ±10 | 48 ±5 | 152 ±12 | 213 ±12 | 183 ±22 | 168 ±15 | 122 ±36 | 219 ±37 | 76 ±55 | 167 ±17 | 98 ±29 | 227 ±41 | 160 ±30 | 182 ±24 | 40,5 |
| M-CSF | * | * | * | * | * | * | * | * | * | * | * | * | * | * | * | * | 2,7 |
| MMP-1 | 3450 ±145 | 5698 ±298 | 4524 ±96 | 3503 ±209 | 4830 ±280 | 6880 ±133 | 5888 ±129 | 5190 ±311 | 4468 ±327 | 6175 ±162 | 6320 ±167 | 5829 ±163 | 3827 ±93 | 4826 ±162 | 4500 ±198 | 3538 ±120 | 27 |
| MMP-9 | * | * | * | * | * | * | * | * | * | * | * | * | * | * | * | * | 27 |
| PDGF-BB | * | * | * | * | * | * | * | * | * | * | * | * | * | * | * | * | 2,7 |
| PLGF | * | * | * | * | * | * | * | * | * | * | * | * | * | * | * | * | 5,4 |
| TGFα | * | * | * | * | * | * | * | * | * | * | * | * | * | * | * | * | 13,5 |
| TGFβ1 | 921 ±180 | 1001 ±140 | 1112 ±91 | 809 ±145 | 1438 ±152 | 1209 ±281 | 1707 ±250 | 1234 ±271 | 1230 ±45 | 1005 ±105 | 1470 ±99 | 974 ±126 | 902 ±67 | 899 ±60 | 1002 ±77 | 560 ±127 | 135 |
| TGFβ3 | * | * | * | * | * | * | * | * | * | * | * | * | * | * | * | * | 54 |
| TIE-1 | * | * | * | * | * | * | * | * | * | * | * | * | * | * | * | * | 675 |
| TIE-2 | * | * | * | * | * | * | * | * | * | * | * | * | * | * | * | * | 27 |
| TIMP-1 | 18220 ±1023 | 20599 ±1189 | 17371 ±1830 | 19593 ±1013 | 23998 ±1585 | 30533 ±812 | 25933 ±982 | 28010 ±2557 | 31738 ±2400 | 34885 ±2970 | 37738 ±1005 | 34885 ±2245 | 33146 ±2822 | 32105 ±2204 | 30677 ±2088 | 35395 ±2231 | 54 |
| TIMP-2 | 29346 ±2684 | 30763 ±3920 | 20642 ±950 | 20394 ±1672 | 21606 ±901 | 29477 ±1009 | 26652 ±970 | 19753 ±1112 | 24830 ±2003 | 28668 ±2206 | 25820 ±1405 | 21190 ±2067 | 22749 ±2230 | 27716 ±3498 | 26235 ±950 | 20781 ±2275 | 27 |
| TNFα | * | * | * | * | * | * | * | * | * | * | * | * | * | * | * | * | 2,7 |
| TNFβ | * | * | * | * | * | * | * | * | * | * | * | * | * | * | * | * | 13,5 |
| TNFRI | 900 ±169 | 1190 ±53 | 1148 ±31 | 1778 ±31 | 1837 ±40 | 1900 ±185 | 2745 ±39 | 2316 ±216 | 1522 ±245 | 1242 ±39 | 3090 ±39 | 2594 ±138 | 777 ±159 | 662 ±28 | 2371 ±401 | 2307 ±130 |  |
| TNFRII | * | * | * | * | * | * | * | * | * | * | * | * | * | * | * | * |  |
| TPO | * | * | * | * | * | * | * | * | * | * | * | * | * | * | * | * | 270 |
| uPAR | 250 ±50 | 198 ±78 | 203 ±22 | 424 ±99 | 2550 ±74 | 3691 ±94 | 3381 ±250 | 2446 ±128 | 3680 ±47 | 3175 ±134 | 4320 ±220 | 2829 ±115 | 2827 ±167 | 3826 ±65 | 4500 ±168 | 2538 ±306 | 54 |
| VEGF-A | 124 ±43 | 132 ±17 | 146 ±9 | 161 ±17 | 150 ±11 | 183 ±16 | 180 ±23 | 204 ±36 | 113 ±17 | 194 ±35 | 201 ±26 | 196 ±33 | 165 ±12 | 199 ±70 | 211 ±54 | 183 ±34 | 13,5 |
| VEGFR2 | * | * | * | * | * | * | * | * | * | * | * | * | * | * | * | * | 13,5 |
| VEGFR3 | * | * | * | * | * | * | * | * | * | * | * | * | * | * | * | * | 27 |
| VEGF-D | 342 ±25 | 287 ±30 | 232 ±44 | 305 ±34 | 396 ±53 | 405 ±55 | 364 ±9 | 483 ±44 | 619 ±101 | 740 ±70 | 509 ±13 | 594 ±48 | 250 ±40 | 247 ±52 | 264 ±39 | 282 ±68 | 54 |
